# Application of Human Genetics to Prioritize Coagulation Cascade Protein Targets for Ischemic Stroke Prevention

**DOI:** 10.1161/STROKEAHA.124.049808

**Published:** 2025-04-06

**Authors:** Iyas Daghlas, Ville Karhunen, Anthony S. Kim, Dipender Gill

**Affiliations:** Department of Neurology, UCSF Weill Institute for Neurosciences, University of California San Francisco (I.D., A.S.K.).; MRC Biostatistics Unit, School of Clinical Medicine, University of Cambridge, United Kingdom (V.K.).; Department of Epidemiology and Biostatistics, School of Public Health, Imperial College London, United Kingdom (D.G.).

**Keywords:** biological specimen banks, embolic stroke, factor V, ischemic stroke, venous thromboembolism

## Abstract

**BACKGROUND:**

While interindividual variations in concentration and function of coagulation cascade proteins are established risk factors for venous thromboembolism (VTE), their associations with arterial ischemic stroke are less well defined.

**METHODS:**

We identified and validated genetic proxies for lifelong, randomized perturbations of coagulation cascade proteins in genome-wide association studies of circulating protein levels (deCODE, n=35 559; UK Biobank, n=46 218) and of VTE risk (81 190 cases and 1 419 671 controls). Study participants were all of European ancestry. We performed 2-sample Mendelian randomization and colocalization analyses to test associations of these genetic proxies with risk of ischemic stroke (62 100 cases and 1 234 808 controls from the GIGASTROKE consortium) and ischemic stroke subtypes, and further contextualized associations with VTE and secondary efficacy and safety outcomes.

**RESULTS:**

We identified genetic proxies for 30 coagulation factors, with cross-trait associations recapitulating canonical coagulation biology. Mendelian randomization and colocalization analyses supported causal associations of genetically proxied levels of 5 proteins with risk of ischemic stroke, with all proteins associating with the cardioembolic stroke subtype: factor XI (odds ratio [OR] of cardioembolic stroke per 1-SD increase, 1.31 [95% CI, 1.19–1.44]; *P*=3.30×10^−8^), high-molecular-weight kininogen (OR, 1.19 [95% CI, 1.09–1.30]; *P*=7.79×10^−5^), prothrombin (OR, 1.83 [95% CI, 1.31–2.57]; *P*=4.20×10^−4^), soluble PROCR (protein C receptor; OR, 0.88 [95% CI, 0.82–0.95]; *P*=6.19×10^−4^), and γ′ fibrinogen (OR per doubling in VTE risk due to lower γ′ fibrinogen levels, 1.44 [95% CI, 1.25–1.66]; *P*=3.96×10^−7^). γ′ Fibrinogen and prothrombin also associated with large artery atherosclerotic stroke, and no proteins were associated with small vessel stroke risk. By contrast, genetic proxies for several coagulation factors (including proteins C and S and factors V and VII) showed selective associations with VTE.

**CONCLUSIONS:**

These data highlight specific coagulation cascade components implicated in ischemic stroke pathogenesis, while identifying proteins with distinct roles in VTE. These findings may inform development of novel anticoagulants and optimize their use in targeted populations with stroke.

Maintenance of hemostasis depends on exquisite control of the concentration and activity of proteins in the coagulation cascade.^1^ Imbalances in procoagulant or anticoagulant protein activity, whether inherited or acquired, can result in venous thromboembolism (VTE). By contrast, although arterial thromboembolism is the recognized mechanism for cardioembolic stroke (CES), the influence of interindividual variation in coagulation cascade proteins on risk of CES and other ischemic stroke subtypes is less well defined.^2,3^ Efforts to identify subgroups of patients with ischemic stroke, beyond those with atrial fibrillation, who derive greater benefit from therapies targeting the coagulation cascade have proven unsuccessful.^4,5^ Accordingly, antiplatelet therapy is the standard preventive treatment for patients with ischemic stroke without atrial fibrillation, though it provides only modest protection against recurrent ischemic stroke.^6^ Understanding how altered coagulation function influences ischemic stroke risk could help identify patients who would benefit more from anticoagulant relative to antiplatelet therapy. More broadly, investigating the relative effects of coagulation cascade proteins on ischemic stroke versus VTE could reveal shared and distinct mechanisms underlying arterial and venous thrombosis. Findings from these investigations may guide discovery and development of anticoagulation therapies with improved efficacy and safety profiles, while informing efficient design of clinical trials through identification of stroke subtype-specific treatment effects.

Addressing these knowledge gaps in how coagulation biology relates to ischemic stroke requires methodologies for causal inference that go beyond conventional associations. The Mendelian randomization (MR) paradigm leverages human genetic data to inform on the causal mechanisms of disease pathogenesis.^7–9^ Germline genetic variants are generally randomly allocated during gametogenesis, which mitigates the confounding by environmental factors that often limits causal inference in traditional epidemiological analyses. This is relevant to circulating coagulation factors, the levels of which are influenced by confounders such as inflammation^10^ and body mass index.^11^ Furthermore, genetic variants are generally unaltered by disease status, thereby mitigating bias due to reverse causality.^8^ Such bias may impact case-control studies investigating the association of coagulation factors and ischemic stroke because the stroke event itself may modify levels of circulating coagulation factors.^12^

In the present work, we leveraged large-scale human genetic data to address fundamental questions about which coagulation cascade proteins influence ischemic stroke risk. First, we identified genetic proxies for these proteins using proteogenomic analyses from the deCODE and UK Biobank (UKB) cohorts, along with case-control genome-wide association studies (GWAS) of VTE (Figure 1). We then analyzed these proxies’ effects on ischemic stroke and its subtypes in the GIGASTROKE consortium, enabling a systematic comparison of their roles in venous and arterial thromboembolic diseases.

**Figure 1.**
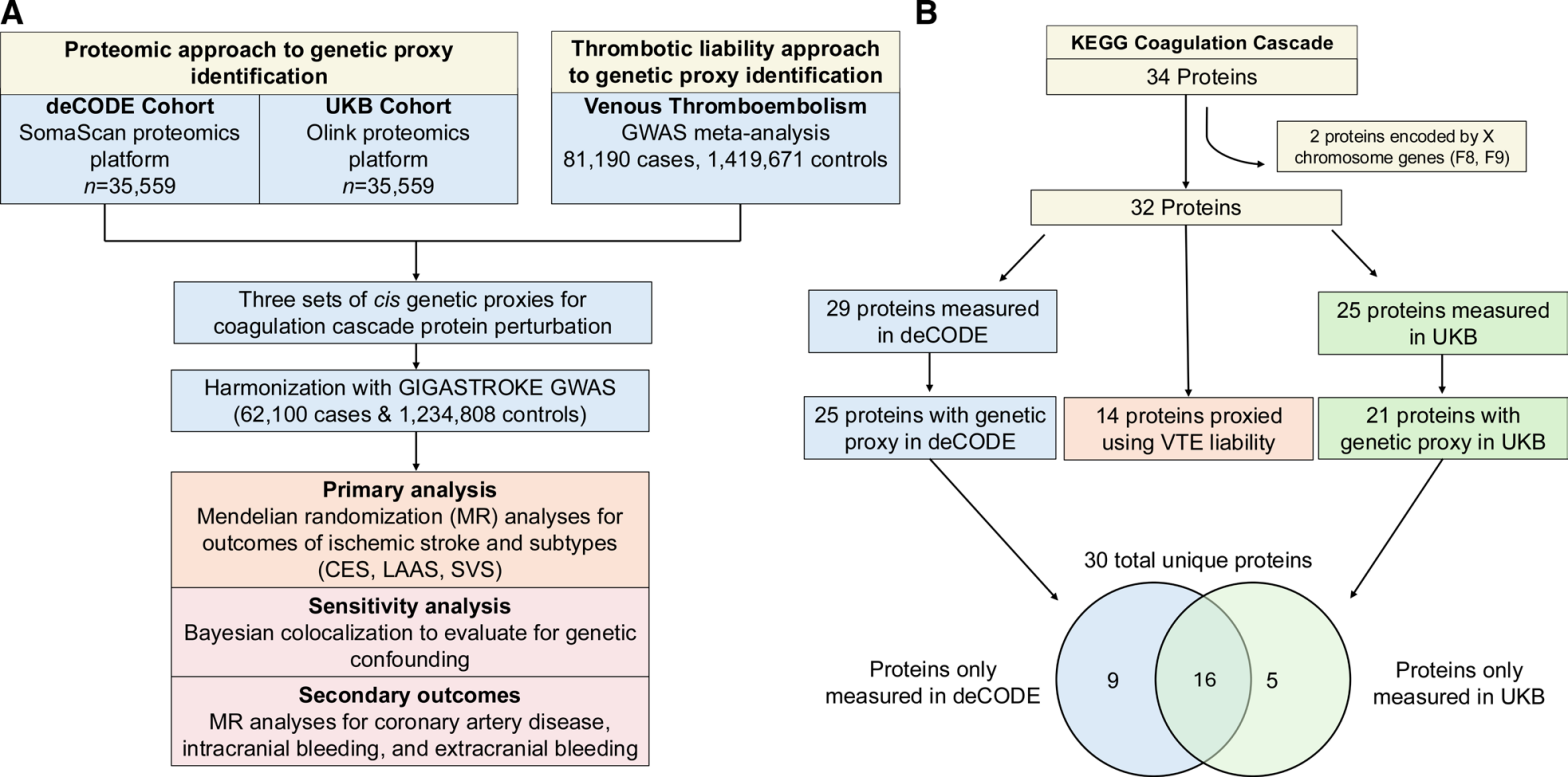
**Flowchart. A,** Study design and (**B**) number of coagulation cascade proteins available for analysis across the 3 methods for genetic proxy selection. All 14 proteins with venous thromboembolism (VTE)–associated proxies were also captured through the proteomic approach, though some variants differed between methods (Table S6). CES indicates cardioembolic stroke; GWAS, genome-wide association study; KEGG, Kyoto Encyclopedia of Genes and Genomes; LAAS, large artery atherosclerotic stroke; MR, Mendelian randomization; SVS, small vessel stroke; and UKB, UK Biobank.

## Methods

### Data Availability

The data used in these analyses are publicly available through GWAS consortium websites and the GWAS catalog. Code used to replicate these analyses is available upon request to the corresponding author.

### Selection of Coagulation Cascade Proteins

A list of coagulation cascade proteins was extracted from the Kyoto Encyclopedia of Genes and Genomes knowledge base (entry hsa04610; Figure 1).^13^ Corresponding genes and their genomic positions were extracted from the GeneCards database (Table S1).^14^ Coagulation factors VIII and IX were excluded from analysis because X chromosome data are typically not available in GWAS summary statistics. For proteins composed of subunits encoded by distinct genes, such as fibrinogen (FGA, FGG, and FGB) and coagulation factor XIII (F13A1 and F13B), we considered the subunit-encoding genes separately when identifying genetic proxies. For example, distinct genetic proxies were identified for factor XIII at F13A1 and F13B. We treated *FGA-FGG* as a single locus due to substantial genetic correlation in this region (Figure S1).

### Genetic Associations With Circulating Coagulation Cascade Proteins and VTE

To identify genetic proxies for levels of circulating coagulation cascade proteins, we leveraged GWAS conducted on plasma protein levels in the deCODE (n=35 559)^15^ and UKB cohorts (n=46 218^16^; Figure 1). Protein levels were measured using the SomaScan platform in deCODE and the Olink platform in UKB.^16,17^ Details regarding the cohorts, methods for measurement of protein abundance, and methods for genetic association analysis are provided in Materials and Methods in the Supplemental Material. The overlap of proteins included on the 2 platforms is presented in Figure 1 and Table S1. Protein measurements from a subset of deCODE samples were analyzed on both the Olink and SomaScan platforms, with cross-platform correlations reported in Table S1.^16^

Genetic variation may alter protein function without impacting protein concentration. Moreover, for membrane-bound proteins, plasma may not be the most relevant tissue compartment. To address these limitations of identifying genetic proxies through associations with circulating protein levels, we also identified genetic proxies for coagulation factor perturbation using genetic associations with VTE (Figure 1). Genetic associations were obtained from a GWAS meta-analysis of 6 cohorts totaling 81 190 VTE cases and 1 419 671 controls of the European ancestry.^18^ VTE cases were predominantly identified using the *International Classification of Diseases* codes for deep vein thrombosis and pulmonary embolism in hospital records. Additional details are provided in the Materials and Methods in the Supplemental Material.

### Genetic Associations With Ischemic Stroke and Secondary Outcomes

Arterial ischemic stroke was the primary outcome of this study. Genetic associations with ischemic stroke were obtained from the GIGASTROKE meta-analysis of 62 100 ischemic stroke cases and 1 234 808 stroke-free controls of the European ancestry.^19^ The mean age of participants across contributing cohorts ranged from 42 to 83 years, with the proportion of female participants varying from 39% to 66%.^19^ Ischemic stroke was subtyped into either CES, large artery atherosclerotic stroke (LAAS), or small vessel stroke according to the Trial of ORG 10172 in Acute Stroke Treatment classification criteria.^20^ Genetic associations with ischemic stroke were adjusted for age, sex, genetic ancestry, and study-specific covariates.^19^ Coagulation cascade proteins supported by both MR and colocalization evidence (colocalization methods described below) for association with ischemic stroke were further evaluated for effects on the secondary efficacy outcome of coronary artery disease (CAD; 181 522 cases and 1 165 690 controls) and on secondary safety outcomes of systemic and intracranial bleeding (Table S2; Materials and Methods in the Supplemental Material).^21–25^

### Identification and Characterization of Genetic Proxies for Perturbation of Coagulation Cascade Proteins

To obtain genetic proxies for coagulation cascade proteins, we first selected *cis* genetic variants positioned within the genes encoding the proteins of interest.^26^ We then filtered the proteomics and VTE GWAS data sets according to the genome-wide statistical significance threshold of 5×10^−8^. We selected variants present across both the exposure and the GIGASTROKE data sets, which implemented a minor allele frequency floor of 0.01. To enhance the functional relevance to coagulation, we further selected variants with at least a nominal association with VTE risk (*P*<0.05). We repeated analyses without this step to ensure that this approach was not missing genetic proxies that selectively influence arterial thrombosis risk. Finally, we identified independent variants using linkage disequilibrium clumping according to an r^2^<0.001 using the 1000 Genomes European panel.^27^ Genetic variants significantly associated with protein levels are henceforth referred to as protein quantitative trait loci (pQTL). This process generated 3 lists of genetic proxies: 1 list of pQTLs from deCODE, 1 list of pQTLs from UKB, and 1 list of genetic proxies from the VTE data set (Figure 1). To contextualize the associations between the identified genetic proxies and with interrelated coagulation cascade proteins, we extracted associations for each genetic proxy with all coagulation factor levels from the deCODE and UKB data sets. This analysis implemented a Bonferroni-corrected *P* value threshold accounting for the total number of unique proteins and variants investigated. To compare pQTL-outcome associations between ischemic stroke and VTE, we plotted *Z* scores for each pQTL association with ischemic stroke (*y* axis) against *Z* scores for the pQTL association with VTE (*x* axis).

### Data Harmonization, MR, and Sensitivity Analyses

Associations of the genetic proxies with ischemic stroke were extracted from the GIGASTROKE and secondary outcome data sets and harmonized by aligning β-coefficients to the same effect allele.^27^ We excluded palindromic variants with minor allele frequencies >0.42, as these could not be unambiguously harmonized.^27^ For single-variant analyses, MR was performed using the Wald ratio method, with SEs estimated using the first-order approximation of the delta method. For analyses involving multiple variants, we used the random-effects, inverse-variance weighted method, which regresses the SNP-outcome association (on the log-odds scale) on the SNP-exposure association with the instrument constrained at the origin. These effects are weighted by the inverse of the SE of the SNP-outcome associations. For MR analyses using pQTLs, the reported estimate represents the association of a 1-SD increase in coagulation factor levels on the odds of ischemic stroke. For analyses utilizing genetic proxies identified through VTE susceptibility, the reported estimate represents the effect on stroke risk of doubling VTE risk via alterations in the relevant coagulation cascade protein.^28^ The statistical significance threshold for the primary analyses investigating ischemic stroke was defined as 0.05 divided by the total number of proteins examined in the analysis.

Valid causal inference from MR analyses requires 3 assumptions: (1) the genetic proxy must be strongly associated with the exposure; (2) the association of the genetic proxy with the outcome is not confounded; and (3) the genetic proxy influences risk of the outcome via perturbation of the exposure of interest and not through alternative pleiotropic mechanisms. Efforts to address these assumptions, including procedures for Bayesian colocalization and conditional analyses, are detailed in Materials and Methods in the Supplemental Material.^29–32^ We identified associations of genetically proxied factor XI and prekallikrein with ischemic stroke risk. These proteins are encoded by the neighboring genes (*FXI* and *KLKB1*), so we performed additional sensitivity analyses to investigate which of these genes is more likely to mediate the regional genetic associations with ischemic stroke (Materials and Methods in the Supplemental Material). All analyses were performed using R, version 4.4.1, using the following packages: TwoSampleMR v0.6.2 R package,^27^ hyprcoloc,^29^ finimom,^33^ and locuszoomr.^34^ This analysis was not preregistered. This article follows the STROBE-MR guidelines (Strengthening the Reporting of Observational Studies in Epidemiology Using Mendelian Randomization).^35^

## Results

### Genetic Proxies for Perturbation of Coagulation Cascade Proteins

Thirty coagulation cascade proteins had at least 1 available genetic proxy (Figure 1; Table S1). We, therefore, implemented a statistical significance threshold of *P*<0.05/30=0.0017 in MR analyses. Genetic proxies identified in each of the 3 exposure data sets are listed in Tables S3 through S5 and are compiled in a single list in Table S6. The genetic proxy list was materially unchanged when omitting the step of filtering pQTLs based on VTE associations.

**Figure 2.**
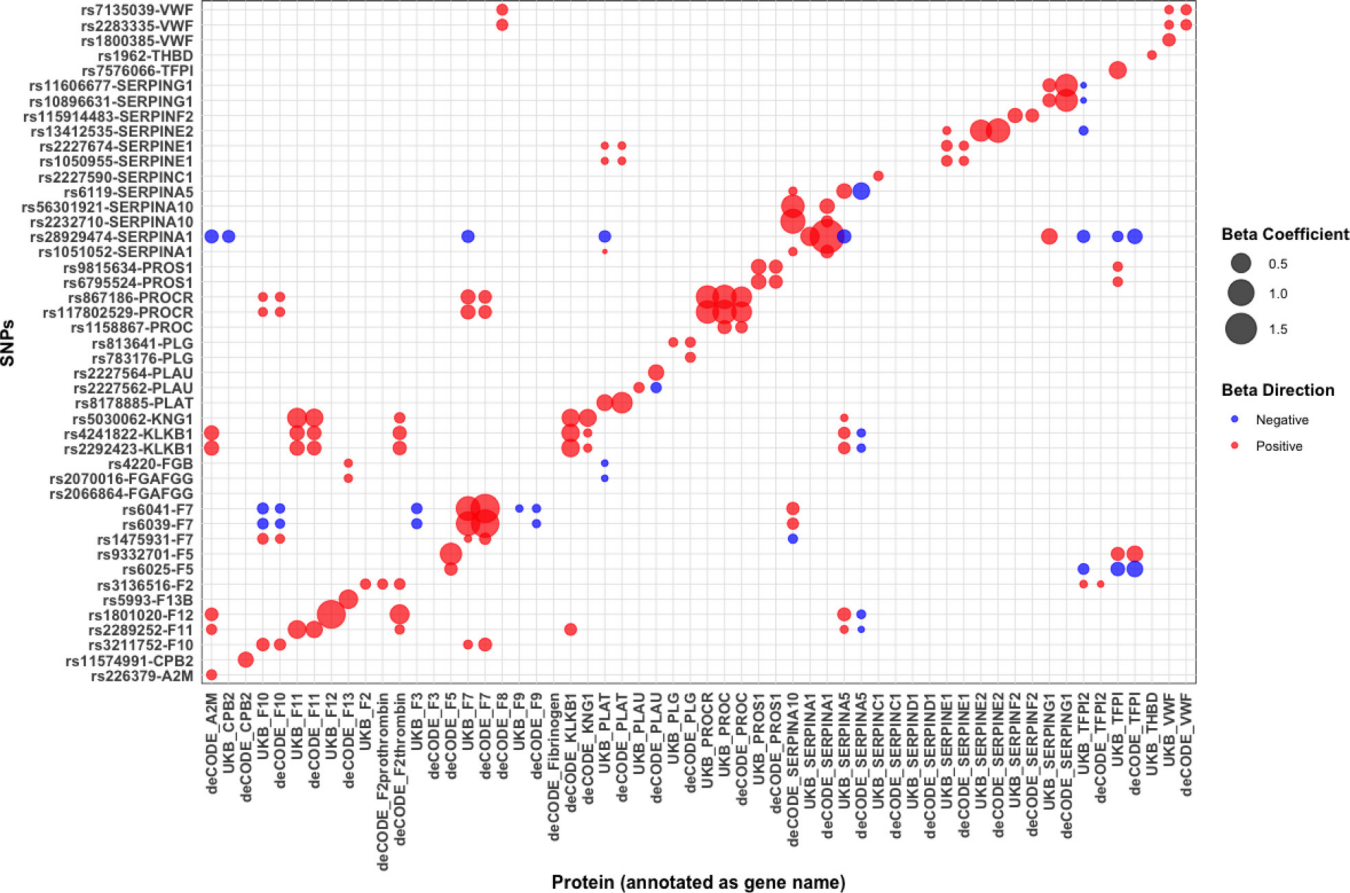
**Association of genetic proxies for coagulation cascade proteins with levels of circulating coagulation factors in deCODE and UK Biobank (UKB).** We only visualized associations with coagulation factors that surpass the Bonferroni-corrected significance threshold accounting for all variants and proteins that were tested (*P*<3.6×10^−5^). The size of the circle represents the magnitude of the β-coefficient for association with the coagulation cascade protein, and the color represents the direction of association (red, positive; blue, negative). All effect estimates are provided in Table S7. SNP indicates single-nucleotide polymorphism.

A library of associations of these genetic proxies with all available coagulation cascade protein levels is presented in Table S7, with statistically significant cross-trait associations displayed in Figure 2 (*P* cutoff, 0.05/1380=3.6×10^−5^). Associations of the genetic proxies with coagulation cascade protein levels recapitulated known biological relationships, including von Willebrand Factor as a carrier protein for factor VIII, protein S as a cofactor for tissue factor pathway inhibitor, and intrinsic pathway proteins as associated with increased thrombin levels (Figure 2). Genetic proxies generally had consistent associations with protein levels across the 2 cohorts. However, variant-protein associations for *PLAU* and *SERPINA5* were in opposite directions across the cohorts, suggesting possible biases due to assay or epitope effects.

### FXI Is Prioritized as the Causal Gene Influencing Ischemic Stroke Risk in the FXI-KLKB1 Gene Region

**Figure 3.**
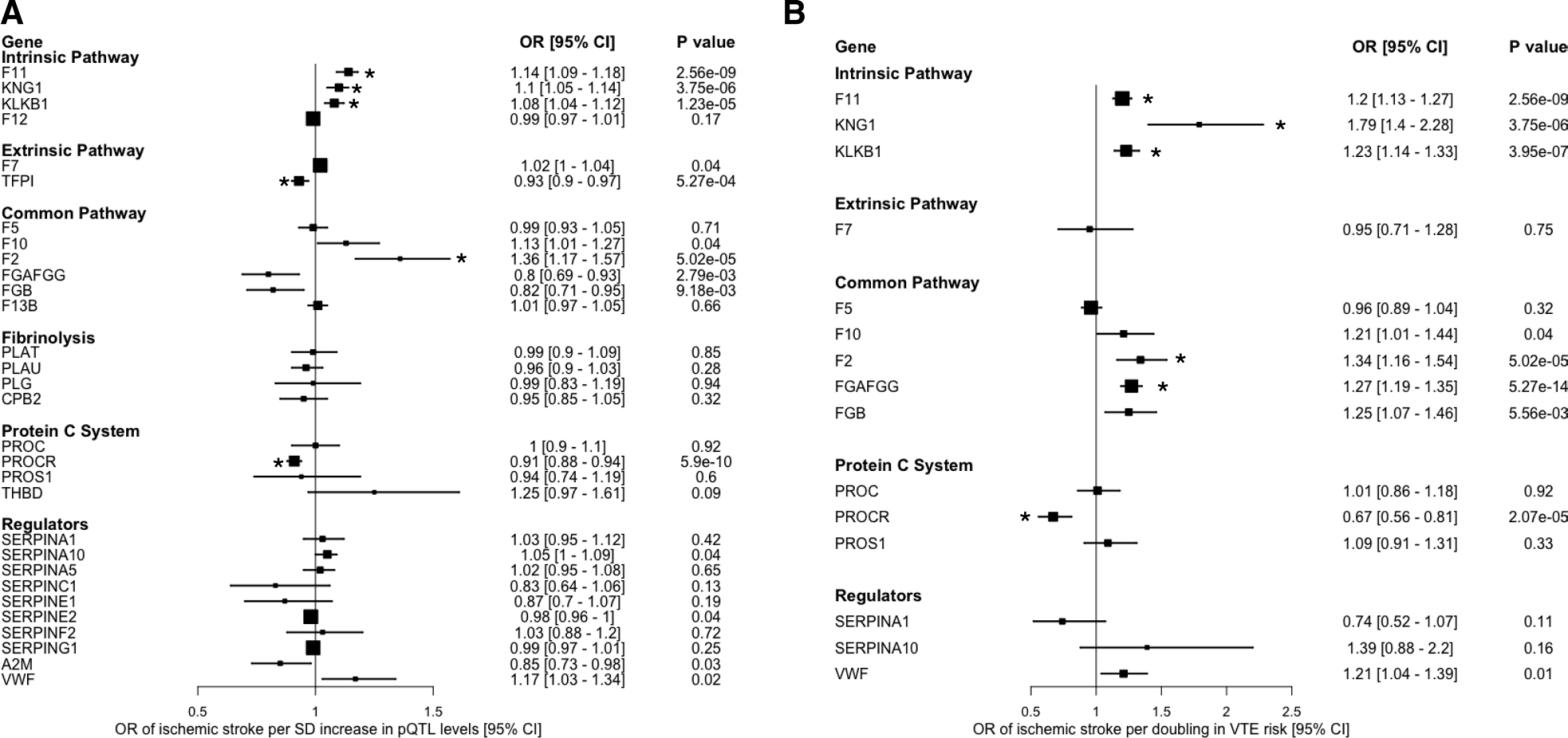
**Mendelian randomization (MR) associations of genetically proxied perturbation of coagulation cascade proteins with ischemic stroke risk.** Genetic proxies were identified through either proteomic associations (**A**) or through venous thromboembolism (VTE) liability (**B**). For visualization purposes, A pools findings from MR analyses using deCODE protein quantitative trait loci (pQTLs) and nonoverlapping pQTLs in UKB. The forest plot depicts associations of genetically proxied coagulation factor levels with risk of ischemic stroke. The black squares represent the point estimate (odds ratio [OR] of stroke per 1-SD increase in protein levels), while the bars represent 95% CIs. The asterisk (*) corresponds to associations that surpass the Bonferroni-adjusted *P* value threshold of 0.0017.

Genetically proxied upregulation of factor XI (encoded by *F11*) and prekallikrein (encoded by *KLKB1*) was associated with increased risk of overall ischemic stroke and CES (Figure 3; Tables S8 through S10). The proximity of *F11* and *KLKB1* led to overlapping regional genetic associations for their respective protein levels. We, therefore, conducted sensitivity analyses to examine which of these 2 proteins more likely mediates the regional genetic associations with ischemic stroke. First, visual inspection of the regional association plots revealed that the ischemic stroke genetic associations aligned more closely with factor XI pQTL associations than with those of prekallikrein (Figures S2 and S3). Second, there was stronger pairwise colocalization evidence with ischemic stroke for *F11* (posterior probability, 0.99) compared with *KLKB1* (posterior probability, 0.80; Table S11). Third, fine-mapping using finimom was consistent with a single ischemic stroke genetic association in this region (posterior probability, 1.00), with the credible set consisting of 4 variants including the lead factor XI pQTL (rs2289252, rs3756011, rs4444878, and rs4253417). Fourth, adjusting the ischemic stroke genetic associations at *FXI-KLKB1* for the lead factor XI pQTL (rs2289252) resulted in no significant residual regional genetic associations (*P*>1.3×10^−4^). Fifth, multivariable MR simultaneously adjusting for genetic predictors of factor XI and prekallikrein levels revealed a significant residual association of factor XI levels with ischemic stroke (odds ratio [OR] of ischemic stroke per 1-SD increase in adjusted factor XI levels, 1.12 [95% CI, 1.08–1.16]; *P*=1.33×10^−10^) and no residual association of genetically proxied KLKB1 levels with ischemic stroke (OR per 1-SD increase in KLKB1, 1.02 [95% CI, 0.99–1.05]; *P*=0.12). Finally, a pQTL specific to factor XI was associated with ischemic stroke risk, whereas a pQTL specific to prekallikrein showed no evidence for association with ischemic stroke risk (Table S12). In secondary analyses, genetically proxied factor XI levels had no evidence for association with risk of CAD or bleeding outcomes (Tables S13 through S18).

### High-Molecular Weight Kininogen, the Carrier Protein for Factor XI, Is Associated With Increased Ischemic Stroke Risk

**Figure 4.**
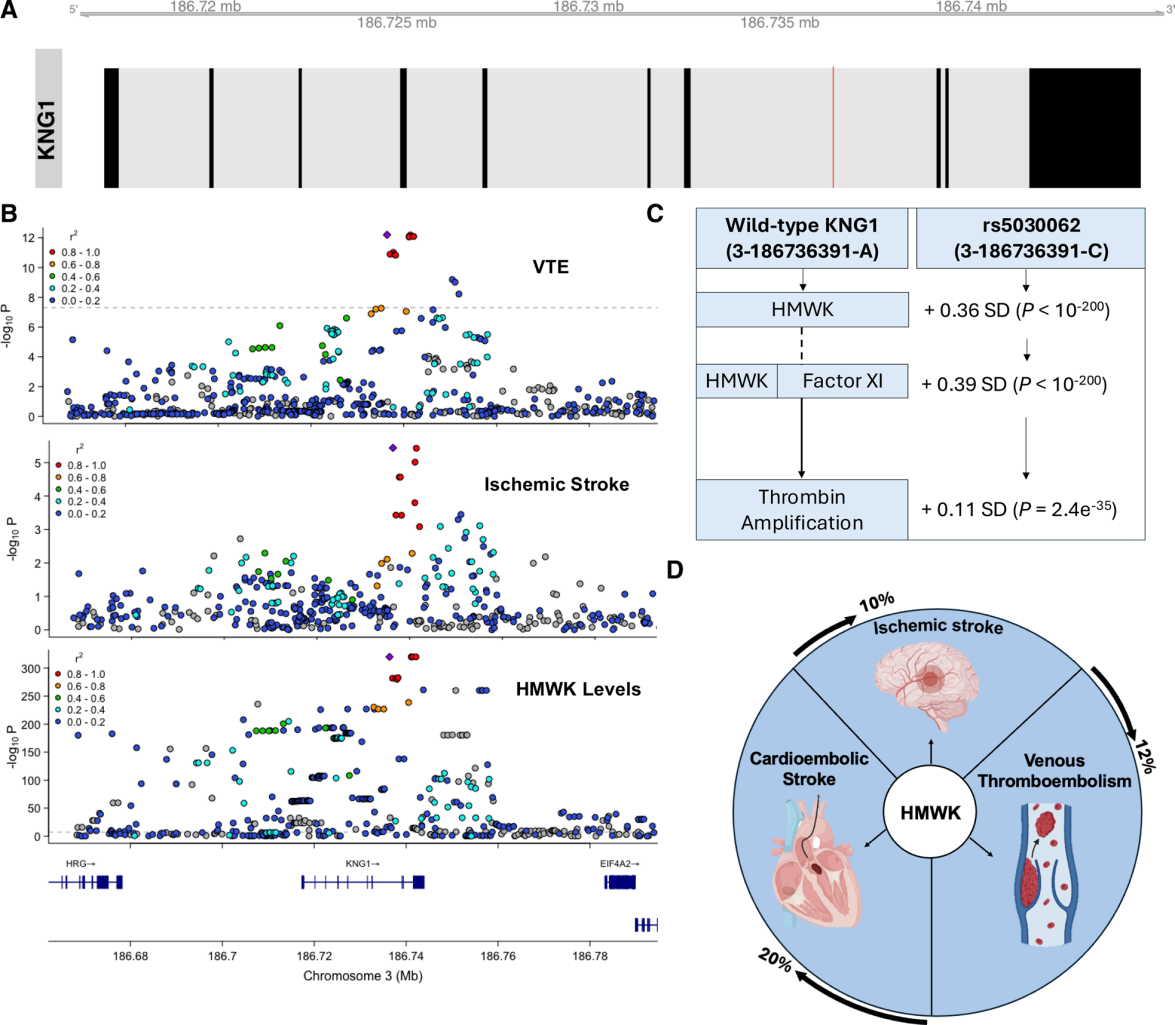
**A genetic proxy for high-molecular-weight kininogen (HMWK) recapitulates its role in factor XI stabilization, thrombin amplification, and increased risk of ischemic stroke, cardioembolic stroke (CES), and venous thromboembolism (VTE). A**, Visualization of the structure of *KNG1*, the gene that encodes HMWK. Black bars represent exons, and gray bars represent introns. Genomic positions are provided according to the GRCh38 genome assembly. The red line marks the genomic position of the rs5030062 variant used to proxy HMWK levels. **B**, LocusZoom plots for regional associations of genetic variants with VTE, ischemic stroke, and plasma HMWK levels. Dots represent genetic variants, and the diamond represents the rs5030062 protein quantitative trait loci (pQTL). The *x* axis represents genomic position in the GRCh38 genome assembly, and the *y* axis represents the −log_10_(*P*) for each variant association with the outcome. Genetic variants are colored according to their linkage disequilibrium estimate with the lead rs5030062 pQTL (using the 1000G European reference panel). **C**, Estimates for associations of the C allele of the rs5030062 variant with HMWK, prekallikrein, factor XI, and thrombin levels. **D**, Visualization of the effects of a genetically proxied 1-SD increase in HMWK levels on the relative risk of VTE, ischemic stroke, and cardioembolic stroke. This figure was created using the BioRender software (BioRender.com).

*KNG1* encodes high-molecular-weight kininogen (HMWK), which stabilizes the majority of circulating factor XI and prekallikrein (Figure 4). Consistent with this biological function, the HMWK-increasing genetic proxy associated with elevated levels of circulating factor XI and prekallikrein, as well as with downstream increases in thrombin levels (Figure 4; Table S7). Genetically proxied upregulation of HMWK was associated with increased risk of ischemic stroke, CES, and VTE (Figures 3 and 4), with strong evidence for pairwise colocalization with ischemic stroke (posterior probability, 0.96). There was no evidence for association with CAD or bleeding outcomes (Tables S13 through S18).

### Prothrombin Is Associated With Increased Risk of Several Ischemic Stroke Subtypes and With CAD

Genetically proxied prothrombin levels were significantly associated with increased risk of ischemic stroke (OR, 1.36 [95% CI, 1.17–1.57]; *P*=5.0×10^−5^) and CES (OR, 1.83 [95% CI, 1.31–2.57]; *P*=4.2×10^−4^) and were nominally associated with the risk of LAAS (OR, 1.63 [95% CI, 1.04–2.57]; *P*=0.03). There was significant evidence for pairwise colocalization between genetic associations with prothrombin and ischemic stroke (posterior probability, 0.72; Figure S4). Genetically proxied prothrombin levels were further associated with increased risk of CAD (OR, 1.17 [95% CI, 1.06–1.29]; *P*=2.38×10^−3^) and with protection from menorrhagia (OR, 0.62 [95% CI, 0.51–0.76]; *P*=3.5×10^−6^). Prothrombin is converted to thrombin through the activity of factor Xa. However, there was no evidence for the factor X genetic proxy being associated with increased thrombin levels (Table S7), and factor X only nominally associated with increased ischemic stroke risk (OR, 1.13 [95% CI, 1.01–1.27]; *P*=0.04).

### Factor V Proxies With Distinct Mechanisms Are Selectively Associated With VTE

The lead VTE proxy (*P*=2×10^−916^) for factor V perturbation was the functional factor V Leiden variant, which was independent of the lead factor V pQTL in deCODE (r^2^=0.0004). Genetic upregulation of factor V, irrespective of genetic mechanism, was selectively associated with VTE but not with ischemic stroke (Figure 3; Tables S8 through S10 and S19).

### Genetic Variants in FGA-FGG That Increase γ′ Fibrinogen Protect Against Multiple Ischemic Stroke Subtypes

The genetic proxy in *FGA-FGG* that was identified using VTE liability (rs2066864) was not associated with total fibrinogen levels in deCODE (Table S7). rs2066864 tags the γ-haplotype 2^36^ and has been associated with levels of γ′ fibrinogen, a fibrinogen isoform with potential anticoagulant effects (A allele associated with lower levels of γ′ fibrinogen and increased VTE risk).^37,38^ Genetically proxied liability to VTE at *FGA-FGG*, corresponding to reduced γ′ fibrinogen levels, was associated with increased risk of ischemic stroke (OR, 1.27 [95% CI, 1.19–1.35]; *P*=5.27×10^−14^), LAAS (OR, 1.45 [95% CI, 1.19–1.76]; *P*=1.83×10^−4^), and CES (OR, 1.44 [95% CI, 1.25–1.66]; *P*=3.96×10^−7^) but not with CAD or bleeding outcomes. There was evidence for colocalization between VTE and ischemic stroke at this locus (posterior probability, 0.82; Figure S1).

### Genetic Proxies for Endogenous Anticoagulants and Risk of Ischemic Stroke

Genetic proxies were identified for 20 proteins with diverse roles in fibrinolysis and anticoagulation, of which 3 had MR evidence for an association with ischemic stroke. Genetically proxied tissue factor pathway inhibitor levels associated with a significant reduction in ischemic stroke risk (OR, 0.93 [95% CI, 0.90–0.97]; *P*=5.27×10^−5^), a nominal reduction in risk of small vessel stroke and LAAS (Table S9), and with reduced VTE risk (Figure 3; Table S19). There was, however, no strong evidence for colocalization at this gene region (posterior probability, 0.22), though the complex, long-range linkage disequilibrium pattern at this locus may have impacted findings from the colocalization technique (Figure S5). Similarly, genetically proxied urokinase levels (using the UKB proxy) associated with reduced risk of CES (OR, 0.48 [95% CI, 0.32–0.74]; *P*=8.00×10^−4^), though colocalization evidence was weak (posterior probability, 0.37). Visual inspection of the regional association plots revealed a nearby confounding signal in *MYOZ1*, which is a known atrial fibrillation risk gene^39^ (Figure S6). This variant (rs2227562) had an opposite direction of effect on urokinase levels in deCODE, suggesting potential epitope effects or assay biases that warrant further targeted investigation (Figure 2).

The p.Ser219Gly missense variant in *PROCR* (protein C receptor; rs867186), identified as a proxy using VTE liability, was in high linkage disequilibrium (r^2^=0.99) with the lead variant associated with soluble PROCR levels in UKB. The G allele of rs867186 was associated with a prothrombotic profile of increased levels of soluble PROCR, protein C, factor VII, and factor X (Figure 2; Table S13). This allele associated with increased VTE risk (Table S15) but unexpectedly associated with reduced risk of ischemic stroke, CES, and CAD (Tables S9, S10, S17, and S18). There was strong evidence for colocalization of genetic associations with soluble PROCR pQTL and ischemic stroke at this locus (posterior probability, 0.99; Figure S7).

### Comparison of Associations of Genetic Proxies With Venous and Arterial Thromboembolism

**Figure 5.**
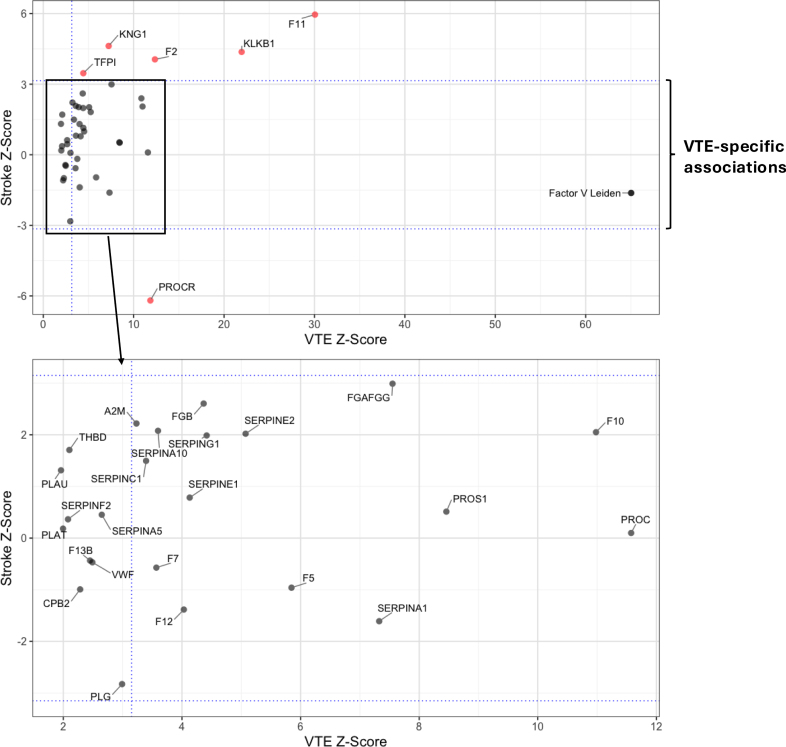
**Comparison of the associations between genetic proxies for coagulation cascade protein levels and risk of ischemic stroke and venous thromboembolism (VTE).** The VTE *Z* score is displayed on the *x* axis, and the ischemic stroke *Z* score is displayed on the *y* axis. The dotted blue lines correspond to a *Z* score of 3.15, which corresponds to the *P* value cutoff of 0.0017 used in the present study. Each dot represents a single variant, with red dots highlighting proxies significantly associated with ischemic stroke. **Bottom** focuses on a narrower range of VTE *Z* scores for clearer visualization.

Figure 5 compares pQTL-VTE associations (reported in Table S19) with pQTL-ischemic stroke associations (Table S8). The 6 pQTLs that were associated with ischemic stroke risk (tissue factor pathway inhibitor, HMWK, prothrombin, prekallikrein, factor XI, and soluble PROCR) were all associated with VTE. In contrast, 15 proteins, including factor V, factor VII, protein C, and protein S, were selectively associated with VTE. This analysis was repeated using coagulation cascade protein proxies identified using VTE liability, which additionally highlighted the *FGA-FGG* proxy as being associated with both VTE and ischemic stroke (Figure S8).

## Discussion

**Figure 6.**
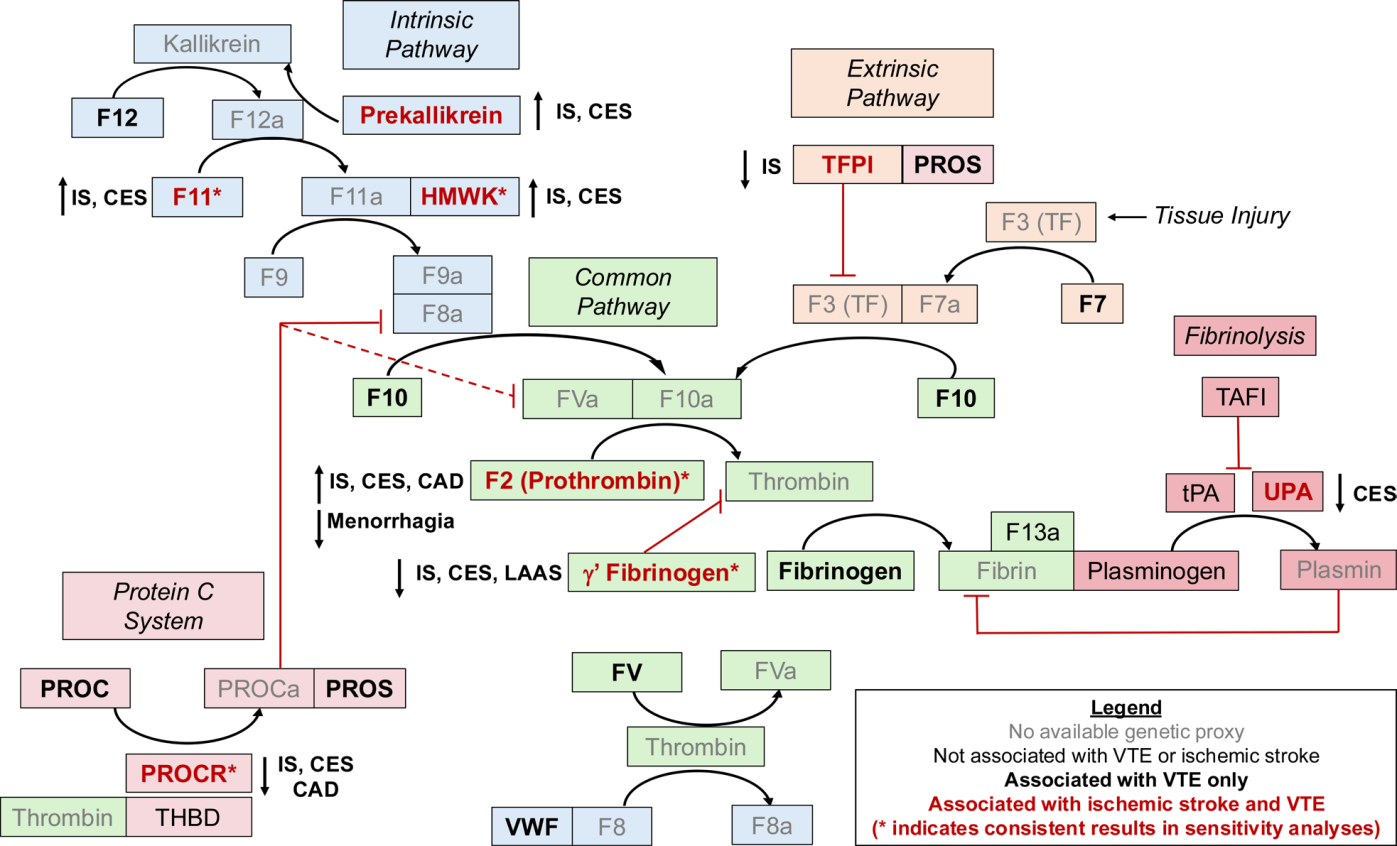
**Contextualization of Mendelian randomization (MR) associations with canonical coagulation cascade pathways.** The visualized canonical pathways were derived from the Kyoto Encyclopedia of Genes and Genomes pathway database and supporting literature and should not be considered exhaustive. Proteins are displayed according to their abbreviated gene name. We excluded Serpin proteins for visualization purposes. The text color corresponds to findings from associations of genetically proxied perturbation of coagulation cascade proteins with ischemic stroke and venous thromboembolism (VTE): gray for proteins without an available genetic proxy; black for genetically proxied proteins not associated with VTE or ischemic stroke; bold black for genetically proxied proteins only associated with VTE; and bold red for genetically proxied proteins associated with ischemic stroke (all of which except for urokinase were also associated with VTE). We note that the anticoagulant mechanisms of γ′ fibrinogen remain incompletely understood. Additionally, the figure depicts the canonical role of endothelial-bound PROCR (protein C receptor); however, our study investigated effects of soluble PROCR, which may act through different mechanisms. CAD indicates coronary artery disease; CES, cardioembolic stroke; FV, factor V; HMWK, high-molecular-weight kininogen; IS, ischemic stroke; LAAS, large artery atherosclerotic stroke; PROC, protein C; PROS, protein S; TAFI, thrombin-activatable fibrinolysis inhibitor; TFPI, tissue factor pathway inhibitor; THBD, thrombomodulin; tPA, tissue-type plasminogen activator; UPA, urokinase-type plasminogen activator; and VWF, von Willebrand Factor. *Consistent results in sensitivity analyses.

This systematic, large-scale genetic analysis identified circulating coagulation cascade proteins that influence arterial ischemic stroke risk, with the strongest evidence provided for prothrombin, factor XI, HMWK, soluble PROCR, and γ′ fibrinogen. These proteins all further associated with VTE, whereas other coagulation factors showed selective associations with VTE alone. These findings, which build on prior investigations,^9,38,40–42^ were enabled by a comprehensive variant selection strategy applied to large GWAS data sets. Figure 6 summarizes these findings in the context of canonical coagulation pathways, supporting several key conclusions.

Our findings enhance the understanding of how coagulation cascade proteins differentially influence risk of ischemic stroke subtypes. Most of the ischemic stroke–associated coagulation cascade proxies demonstrated specific associations with CES, suggesting that interindividual variation in arterial thrombosis tendency contributes to CES risk over-and-above atrial fibrillation liability. Future research should evaluate whether including this dimension of thrombotic liability could enhance stroke risk stratification in atrial fibrillation. A subset of the coagulation cascade proxies further associated with LAAS risk, supporting the contribution of specific coagulation pathways to risk of carotid plaque rupture. By contrast, we found no evidence for an association between any genetically proxied coagulation cascade proteins and small vessel stroke risk. This null finding warrants investigation into the relative efficacy of anticoagulation versus antiplatelet therapy for preventing small vessel stroke.

We uncovered several novel lines of genetic evidence specifically implicating components of the intrinsic and common coagulation pathways in ischemic stroke risk. While the genetic association of factor XI with ischemic stroke has been previously described, there exists uncertainty as to whether this association is driven by the neighboring *KLKB1* gene.^40^ All lines of evidence from our analysis prioritized *F11* over *KLKB1* as the causal gene in this region. We report a novel association between genetically proxied levels of HMWK, the carrier protein for factor XI, and ischemic stroke risk, providing additional evidence that factor XI is an efficacious drug target for ischemic stroke prevention.^43^ Conversely, HMWK itself is less likely to be an appropriate target due to its pleiotropic role in bradykinin generation.^43^ The genetic proxy for elevated prothrombin levels associated with increased thrombin levels and increased risk of ischemic stroke and CES, consistent with the established clinical efficacy of direct thrombin inhibitors for CES prevention.^44^ There was also evidence for an association between genetically proxied prothrombin levels and risk of LAAS and CAD, supporting additional investigation into the efficacy of this drug target for the prevention of stroke due to atherosclerotic mechanisms.^45^ These effects may, in part, be attributable to pleiotropic roles of thrombin in platelet activation in addition to its canonical role in the coagulation casacade.^1^ The inverse association between genetically proxied prothrombin levels and menorrhagia risk suggests a sex-specific evolutionary advantage to prothrombotic variation at this gene. This hypothesis, while not the focus of the present study, could be tested in greater depth in future investigations of related phenotypes such as postpartum bleeding.^46^

Despite the established role for factor X in conversion of prothrombin to thrombin, the factor X genetic proxy did not associate with thrombin levels. This suggests that compensatory biological processes may offset effects of this variant on arterial thromboembolism. We accordingly observed only a nominal association of genetically proxied factor X levels with risk of ischemic stroke. Larger genetic studies are required to replicate this nominal finding and to investigate low-frequency variation in this locus. Additional experimental work is also needed to determine the mechanisms underlying the opposing associations of genetically proxied soluble PROCR levels with VTE and arterial thrombosis and the protective association of genetically proxied γ′ fibrinogen with ischemic stroke and VTE.^47^ While there was evidence for protective associations between circulating tissue factor pathway inhibitor and urokinase levels and ischemic stroke, evidence for colocalization was weak, and this finding requires further investigation in larger data sets.

The coagulation cascade proteins that associated with ischemic stroke were further associated with VTE, challenging the notion of a strict dichotomy in the pathogenesis of thrombosis in venous and arterial beds. By contrast, several coagulation cascade proteins, including protein C, protein S, and factors V and VII, were selectively associated with VTE. This contrasts with findings from a prior study that reported protein C as being causally related to ischemic stroke risk on the basis of an MR analysis using p.Ser219Gly in *PROCR* as a *trans* proxy. However, this variant affects PROCR, which has multiple biological functions beyond protein C signaling, making it unsuitable as a genetic proxy for protein C effects; this highlights the analytic advantage of using *cis*- rather than *trans*-acting genetic variants. The null association of factor V Leiden with ischemic stroke is notable and suggests limited clinical utility of testing for this variant as part of an ischemic stroke evaluation.^2^ The mechanisms underlying specificity of certain coagulation pathways to VTE remain speculative but may relate to differences in shear forces in the arterial and venous systems.^3^ From a therapeutic perspective, these findings suggest that only a limited set of coagulation targets would be expected to prevent ischemic stroke over-and-above VTE.

This study has several notable strengths. The MR study design mitigates bias due to confounding and reverse causality, thereby strengthening confidence in the causal nature of the reported associations. Findings from MR analyses were further supported by colocalization analyses. We implemented a stringent process for selecting genetic proxies that triangulated across multiple approaches and maximized statistical power by leveraging the largest available GWAS data sets. Several proteins, such as prothrombin, factor XI, HMWK, and soluble PROCR, had identical or highly correlated (r^2^=0.99) proxies identified across the 3 exposure data sets, increasing confidence in the relevance of the variant to perturbation of the target protein. Finally, the investigation of ischemic stroke subtypes permitted granular investigations into stroke pathophysiology that revealed subtype-specific associations, with implications for understanding disease mechanisms and informing targeted therapeutic strategies.

This study also has several limitations. First, we cannot exclude the possibility that genetic proxies influence ischemic stroke risk through pathways independent of the coagulation factor of interest or that genetic associations are confounded. Second, our proteomic data only encompassed measurements of protein levels. Future studies incorporating functional coagulation assays would enable further validation of genetic proxies for coagulation cascade proteins and may identify additional variants operating through distinct biological mechanisms. Third, our approach of only considering genetic variants within the genes of interest, while minimizing inclusion of invalid proxies, may have excluded relevant neighboring regulatory variants. Fourth, there was a small amount of sample overlap between the exposure and outcome data sets (eg, inclusion of deCODE in VTE and ischemic stroke GWAS). The impact of this sample overlap on our findings is likely minimal due to our use of strong genetic proxies that minimize weak instrument bias.^48,49^ Fifth, this analysis assumes a linear relationship between coagulation factor levels and ischemic stroke risk, which may not hold for coagulation factors that have nonlinear biological effects.^50^ This limitation may account for the null effects of some genetic proxies on ischemic stroke and bleeding outcomes. Identifying such nonlinear relationships requires more complex modeling approaches using individual-level data in future studies. Sixth, genetic variants that induce lifelong alterations in coagulation factor levels or function may result in compensatory mechanisms that modify protein associations with ischemic stroke risk. These adaptive biological responses may impact the magnitude of genetic associations, limiting direct translation to shorter-term pharmacological interventions. Seventh, the minor allele frequency floor of 0.01 implemented in GIGASTROKE precluded analysis of low-frequency variation. Eighth, we did not have available data to test for heterogeneity by the presence or absence of right-to-left cardiac shunts, by atrial fibrillation status, or by sex. Finally, future investigations should repeat these analyses in non-European populations to improve generalizability of these findings.

In conclusion, our systematic genetic investigation identified several proteins with potentially causal effects on ischemic stroke risk and clarified their distinct contributions to arterial versus venous thrombotic risk. These findings lay a foundation for multiple therapeutic hypotheses that merit further exploration.

## ARTICLE INFORMATION

### Acknowledgments

The authors are grateful to Leena Suleiman for helpful comments on an early draft.

### Sources of Funding

Dr Gill was supported by Medical Research Council grant MR/X0113721/1 and the British Heart Foundation Center of Research Excellence (RE/18/4/34215) at Imperial College London. Dr Kim reports grant support from the National Institutes of Health, American Heart Association, and Patient-Centered Outcomes Research Institute.

### Disclosures

Dr Gill is the Chief Executive Officer of Sequoia Genetics, a private company that works with investors, pharma, biotech, and academia by performing research that leverages genetic data to help inform drug discovery and development. The other authors report no conflicts.

### Supplemental Material

Supplemental Materials and Methods

Tables S1–S19

Figures S1–S8

STROBE-MR Checklist
